# Can treatment of malaria be restricted to parasitologically confirmed malaria? A school-based study in Benin in children with and without fever

**DOI:** 10.1186/1475-2875-9-104

**Published:** 2010-04-21

**Authors:** Jean-François Faucher, Patrick Makoutode, Grace Abiou, Todoégnon Béhéton, Pascal Houzé, Edgard Ouendo, Sandrine Houzé, Philippe Deloron, Michel Cot

**Affiliations:** 1 Institut de Recherche pour le Développement (IRD); UMR 216: Mother and Child face to tropical infections, Paris, France; 2 Departement of Infectious Diseases, Besançon University Medical Center, 25030 Besançon cedex, France; 3 Regional Public Health Institute of Ouidah, Benin; 4 Department of Parasitology, Faculty of Health Sciences, Cotonou, Benin; 5 Saint-Louis Hospital biochemistry laboratory, Paris, France; 6 Université Paris Descartes, Paris, France; 7 Laboratory of Parasitology, AP-HP Bichat-C. Bernard Hospital, Paris, France

## Abstract

**Background:**

Applying the switch from presumptive treatment of malaria to new policies of anti-malarial prescriptions restricted to parasitologically-confirmed cases is a still unsolved challenge. Pragmatic studies can provide data on consequences of such a switch. In order to assess whether restricting anti-malarials to rapid diagnostic test (RDT)-confirmed cases in children of between five and 15 years of age is consistent with an adequate management of fevers, a school-based study was performed in Allada, Benin.

**Methods:**

Children in the index group (with fever and a negative RDT) and the matched control group (without fever and a negative RDT) were not prescribed anti-malarials and actively followed-up during 14 days. Blood smears were collected at each assessment. Self-medication with chloroquine and quinine was assessed with blood spots. Malaria attacks during the follow-up were defined by persistent or recurrent fever concomitant to a positive malaria test.

**Results:**

484 children were followed-up (242 in each group). At day 3, fever had disappeared in 94% of children from the index group. The incidence of malaria was similar (five cases in the index group and seven cases in the control group) between groups. Self-medication with chloroquine and quinine in this cohort was uncommon.

**Conclusions:**

Applying a policy of restricting anti-malarials to RDT-confirmed cases is consistent with an adequate management of fevers in this population. Further studies on the management of fever in younger children are of upmost importance.

## Background

Presumptive treatment of fevers with chloroquine has been formerly promoted in sub-Saharan malaria endemic areas. Treatment with anti-malarials restricted to parasitologically-confirmed cases is now promoted [1,2]. Discrepancies between laboratory results and anti-malarials prescriptions show that healthcare providers may not trust blood smear results in routine practice [3-5]. Malaria rapid diagnostic tests (RDTs) have been adopted as public heath policy in several African countries, but some discrepancies between RDT results and anti-malarial prescriptions are an unresolved critical issue [6-10].

Restricting anti-malarials to parasitologically-confirmed cases has been recommended in many countries only for children of five years of age or above (provided these children are less at risk for severe malaria); this discrepancy between age groups in terms of guidelines [1] on the management of fevers raises questions on the rationale of this strategy [11,12]: if such a management of uncomplicated fevers is reliable, why is it not applied also to small children?

Providing that no diagnostic tool is perfect, collecting prospectively data in order to show that applying recent policies on the management of fever is safe and feasible (i.e. does not lead to hospitalizations or death related to malaria, and does not frequently lead to undiagnosed cases of uncomplicated malaria) is now very important.

The main objective of this school-based, prospective cohort study was to collect clinical, parasitological and pharmacological (self-medication) data in a pragmatic approach in order to see whether, regardless of cost-effectiveness issues, applying the algorithm on the management of fevers is consistent with adequate clinical care.

## Methods

### Ethical statement

Ethical clearance was obtained from the Faculty of Medicine ethics committee, Benin National University, Cotonou, and from the Deontology and Ethics Committee from the Institute of Research for Development. Written informed consent was obtained from all children or their parents/guardians before enrolment in the study.

This study was designed to measure the incidence of malaria attacks during a two-week follow-up period in two populations of patients. First, an index group (with fever) constituted by schoolchildren attending the school nursery for fever and not diagnosed malaria, based on a negative HRP2-based RDT. Second, a control group (without fever) constituted of apparently healthy schoolchildren paired with index cases on gender, age, week of inclusion and malaria status (i.e. negative HRP2-based RDT).

Hence, the objective of the study was to assess whether applying the algorithm of management of fevers in the school setting is consistent with adequate management of fevers, in the way it does not lead to a high number of undiagnosed (and thus untreated) malaria attacks, and by comparing the incidence of malaria attacks between the two groups (with and without fever).

### Study site

This study was performed from February through June 2008 (rains started in April) in four schools, located at Allada, southern Benin, where malaria transmission is intense and perennial [13]. Altogether, the four study schools had a total of about 2,600 pupils of five years of age and above.

### Performance of the study

Children attended school nurseries on their own, or led by their parents/guardians. Care was provided for any child from a school participating to the study. Children from the index group were included and actively followed only if individual informed consent was obtained (within 24 hours from the initial visit) from a parent or guardian. Asymptomatic children were screened for the control group on gender, age, week of inclusion. The first child with a negative RDT and for whom an informed consent was obtained was included in the control group. Asymptomatic children (screened for inclusion into the control group) and with a positive RDT were advised to quickly attend at the school nursery as soon as symptoms of malaria would appear. Children treated for malaria within the prior month were not included in either group.

All children were examined by a study nurse. Children with danger signs were referred to a health centre. Children attending the school nurseries were managed for fever only if fever was stated (tympanic temperature at 37.8°C or above [14]) or if an history of fever in the preceding 24 hours was reported either by the child or his parents/guardian. For each schoolchild managed for fever, a RDT (Paracheck^®^, Orchid Diagnostics) [15] was performed and children were given artemether-lumefantrine if the RDT result was positive, according to national guidelines in this age group. At enrolment and during follow-up, medications with anti-malarial activity for the treatment of non-malarial illnesses were avoided when acceptable alternatives were available.

Follow-up visits were on day 3, 7 and 14. A blood smear was collected at enrolment and at each scheduled visit. An additional blood spot for pharmacological data was collected for 100 children in each group on day 0 and day 14. When fever was reported and/or stated at a follow-up visit, a RDT was performed.

### Laboratory analysis

RDTs were read by the study nurse, as recommended by manufacturer 15 minutes after it was performed. A Giemsa-stained thick blood film was prepared at inclusion and at each follow-up visit. Microscopy examination was done retrospectively (patients were managed according to RDT results in terms of anti-malarials prescriptions and assignment to the index group or the control group) and blinded to the patient's identity. Parasite density was determined according to the number of parasites per 200 white blood cells (WBC), and assuming a total WBC count of 8,000/μL. Slide quality control was done by masked re-reading of 10% of slides, selected randomly.

On day 0 and day 14 in the 100 first children from each group, whole blood was sampled on filter paper spots and dried at room temperature. Using these dried blood spot (DBS), chloroquine (and monodesethylchloroquine) and quinine were detected by high pressure liquid chromatography with UV detection to 254 nm using ammonium acetate (40 mM, pH = 5.5)/acetonitril (85/15% v/v) [16].

In order to find an explanation to false negative RDT results, the exon 2 of *pfhrp2 *gene was amplified from genomic DNA in corresponding isolates, by PCR using the specific primer pairs *Pfhrp2-F1 *and *Pfhrp2-R1 *[17].

### Statistics

Parametric tests (Pearson's chi-square and chi-square for the comparison of crude rates [18] and non-parametric (Fischer) tests were used.

## Results

**Figure 1:**
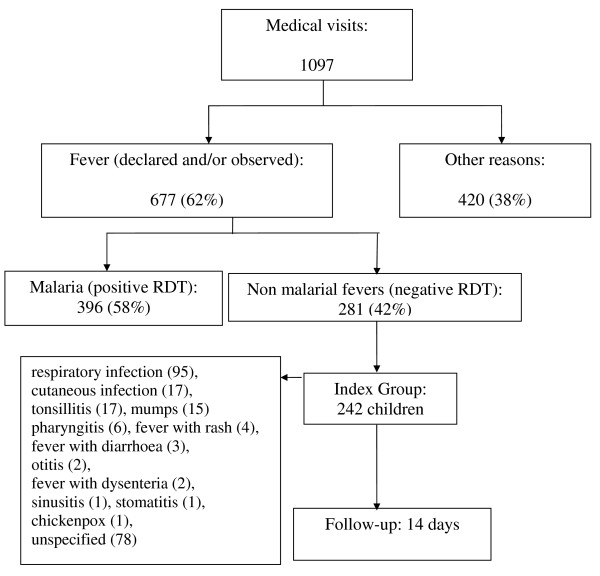
**Profile of visits made to the study clinic**.

Baseline data are presented in Figure 1 and Table 1. Half of the children in the index group (124/242) had a temperature at or above 37.8°C and 29% (71/242) had a temperature at or above 38°C.

**Table 1:** Characteristics of children, matched on gender, age, week of inclusion and rapid diagnostic test status.

Children characteristics	IG	CG	p value
Sex, no. male/female	104/138	105/137	0.93
Age, years; n = 242	9,1 (2.5)	9,1 (2.5)	0.95
Body temperature, °C; n = 242	37.7 (0.7)	37.1 (0.3)	< 10^-5^
Positive blood smear, day 0	12 (including 1 *P. malariae*); n = 242	3 (including 1 *P. malariae*); n = 236	0.02
Bednet use	54; n = 234	46; n = 238	0.32
Chloroquine detection day 0	13; n = 100	6; n = 99	0.1
Chloroquine detection day 14	7; n = 98	8; n = 98	0.79
Quinine detection day 0	1; n = 100	0; n = 99	_
Quinine detection day 14	0; n = 99	0; n = 98	_

Quantitative data are mean (SD). SD: Standard deviation; IG: index group; CG: control group.

A total of 494 RDTs were performed in apparently healthy children and 213 (43%) were positive. Among the 281 children who had a negative RDT, consent was obtained in 242 for their participation into the study. Most of them (62%) were prescribed an antibiotic and paracetamol was widely used.

The median delay between inclusions in the index group and in the control group was 0 day. In the index group, blood smears were positive in 12 children (two had a parasitaemia above 1,000 trophozoites: 3,572/μL and 13,605/μL; one had a *P. malariae *parasitaemia). The exon 2 of *pfhrp2 *gene was amplified with success ruling out the hypothesis of *pfhrp2 *gene deletion to explain false negative results.

### Follow-up of index group children

Children of the index group were followed up a total of 465 person-weeks (Table 2). One child withdrew consent on day 3. Three children withdrew because of declared self-medication at home with an ACT. Six children were lost to follow-up. At day 3, fever had disappeared in 94% of children from the index group. Thirty-one episodes of persistent or recurrent fever were observed in 30 children during follow-up, 14 of them at or before day 3. An alternative diagnosis for fever was made during follow-up in three cases (all treated with antibiotics with no anti-malarial activity): two respiratory infections (day 1 and day 14) and one tonsillitis (day 13).

**Table 2:** Malaria cases observed during follow-up in the whole cohort

Case definition (case numbers)	IG (242 patients)	CG (242 children)	p value
Fever + RDT	5	7	0.56
Fever + blood smear	6	5	0.76

IG: index group; CG: control group.

When taking account solely cases of fever with a positive RDT, five malaria attacks were recorded (one at day 2, two at day 3, one at day 7 and one at day 14) in five index group children. No malaria severity or danger sign was observed. At enrolment, two (malaria diagnosed at day 2 and day 14) had a positive blood smear (3,573/μL and 171/μL respectively).

Among the 12 children who had a positive blood smear at baseline, three had malaria attacks (two when taking into account solely cases of fever with a positive RDT) during the follow-up. The nine other children never presented with fever during the follow-up; parasitaemia spontaneously cleared in eight of these and remained persistently low (<100/μL) in one child.

### Follow-up of control group children

Children of the control group have been followed-up a total of 477 person-weeks (Table 2). Three children were lost to follow-up (one at day 3 and two at day 7), and no child withdrew consent. Nineteen episodes of fever were recorded during follow-up in 19 children, three of them at or before day 3. A new diagnosis was established in four cases (four respiratory infections), all treated with antibiotics with no anti-malarial activity. RDT were not performed in five of the 19 episodes of fever, but concomitant thick blood smears were negative.

When taking into account solely cases of fever with a positive RDT, 7 malaria attacks (1 at day 3, 2 at day 7, 2 at day 10, 1 at day 11 and 1 at day 14) were recorded in 7 children. At enrolment of these seven children, none had a positive blood smear. Overall, one malaria attack was not confirmed by microscopic examination. The clinical picture in this case was stomatitis. Rates of self-medication did not differ between groups (Table 1).

## Discussion

After decades of presumptive treatment strategies, the restriction of anti-malarials to parasitologically-confirmed cases is necessarily difficult. Unfortunately, few studies have dissected the consequences of applying the algorithm of management of fever. Treating any fever in children under five years of age with anti-malarials was recommended in the study area. Therefore, only children from the age of five years were included. The schoolchildren in the study area were semi-immune and occurrence of severe malaria was unlikely in this population, however uncomplicated malaria may serve as a proxy for severe malaria in less immune persons. Njama Meya *et al *[19] used blood smears as a unique diagnosis tool to confirm malaria, and subsequently passively followed-up children (including children under the age of 5 years). They found very few (0.8%) febrile illnesses related to malaria in the seven days subsequent to visits for fever, in children who were not treated with anti-malarials. Furthermore, these cases were uncomplicated and successfully treated. The incidence of malaria in the seven days following a negative smear in febrile patients, was significantly lower than the incidence of malaria in their entire cohort [19]. Msellem *et al *[20] used RDTs in a situation of moderate to low transmission intensity and conducted a cross-over clinical trial of symptom-based clinical diagnosis versus clinical diagnosis plus RDT. They followed patients actively at day 14 and found a reduction of both perceived unsuccessful care and anti-malarial prescription in the clinical diagnosis plus RDT arm. The findings of these two studies support the policy of restricting anti-malarials to parasitologically-confirmed cases.

More malaria cases during follow-up were found in the control group, but similar malaria incidence in the two groups. Treating with an anti-malarial a child with fever and a negative RDT seems not more useful than giving the same treatment to an apparently healthy child. This conclusion is based on the management of fever of 242 children, representing a patient flow of several months in many health care facilities. Therefore, this study might be underpowered to compare malaria incidence.

Malaria was the most frequent diagnosis (36% of all medical visits) and no attack of severe malaria was observed during follow-up, as this was expected in this population of children. Since fever had disappeared in 94% of children from the index group, at day 3, educating mothers to bring back their child after two or three days in case of persistence of fever is, therefore, feasible: only a small minority of mothers would have to come back to the health care facility. Malaria attacks were not rare among children with persistent or recurrent fever (five out of 30) in the index group and in this case testing again with a RDT when fever persists, rather than referring patients directly, should be discussed.

The baseline status of the two groups was the negativity of RDT. Interestingly, microscopically detectable parasitaemia at baseline, although rare in both groups, was more frequent in the index group. Differences in terms of self-medication can hardly explain these parasitological differences between groups. Because chloroquine and quinine were the most commonly drugs used for self-medication in the study area, they were chosen as markers of self-medication, though other anti-malarials like SP, were widely available at the time of the study.

As in previous studies [19,20], undiagnosed malaria attacks at enrolment have lead to a few visits for persistent or recurrent fever and once malaria was finally diagnosed, it was uncomplicated and successfully treated. Two negative RDTs in children with a parasitaemia above 1,000/μL were observed. The inability of a diagnosis tool to detect all malaria attacks may certainly be an obstacle to full adherence of medical care providers to the algorithm on the management of fevers. Providing that RDTs are frequently positive in apparently healthy children in endemic areas, life-threatening non-malarial causes of fever may be concomitant to a detectable parasitaemia and, therefore, a positive RDT may also be misleading. Whether or not anti-malarials are prescribed at a medical visit, the persistence of fever after two days must always lead patients to present again to the outpatient clinic to reassess the diagnosis.

Self-medication with quinine and chloroquine was uncommon in this cohort of children who had access to care, and it did not differ significantly between groups, though there was a trend to more self-medication with chloroquine at baseline in the index group.

## Conclusions

Applying fully a policy of treatment of malaria restricted to RDT-confirmed malaria cases appears to be consistent with adequate management of fevers in this population. Giving an anti-malarial treatment to a child with fever and a negative RDT or to an apparently healthy child with a negative RDT is equally useful. Further studies on the management of fever in younger children are of upmost importance.

## Competing interests

The authors declare that they have no competing interests.

## Authors' contributions

JFF designed the study, led field work interpreted the results and drafted the manuscript. PM, GA and TB participated in data collection. GA contributed with data collection in the field. SH performed molecular assays. EO designed the study. PH performed pharmacology assays. PD designed the study and critically read the manuscript, MC designed the study, performed the statistical analyses and critically read the manuscript. All authors read and approved the manuscript.
